# High‐Purity Single Photon Extraction from Diamond Nitrogen‐Vacancy Centers in Low‐Numerical‐Aperture Optical Systems via Defect‐Selective Metalens Printing

**DOI:** 10.1002/smll.202510745

**Published:** 2025-11-02

**Authors:** Minseok Jeon, Moohyuk Kim, Nu‐Ri Park, Yeeun Choi, Junghyun Lee, Chulki Kim, Sang‐Wook Han, Dongyeon Daniel Kang, Seung‐Woo Jeon, Myung‐Ki Kim

**Affiliations:** ^1^ KU‐KIST Graduate School of Converging Science and Technology Korea University Seoul 02841 Republic of Korea; ^2^ Center for Quantum Technology Korea Institute of Science and Technology Seoul 02792 Republic of Korea; ^3^ Division of Nanoscience and Technology KIST School Korea University of Science and Technology (UST) Seoul 02792 Republic of Korea; ^4^ Department of Integrative Energy Engineering College of Engineering Korea University Seoul 02841 Republic of Korea

**Keywords:** diamond NV centers, metalenses, nanophotonics, quantum interface, single‐photon emission, transfer printing

## Abstract

Solid‐state quantum emitters, such as nitrogen‐vacancy (NV) centers in diamond, offer a promising route toward scalable quantum technologies. However, their random spatial distribution and inherently broad dipole emission severely hinder high‐purity single‐photon extraction—particularly in low‐numerical‐aperture (NA) optical systems, which are essential for compact and scalable quantum photonic systems. Here, a defect‐selective metalens integration approach is presented that allows precise and efficient single photon collection from individual NV centers, even under ultra‐low‐NA optical systems. Using an in situ transfer‐printing process, high‐purity silicon dioxide metalenses are stamped precisely and deterministically onto selected NV centers located 25 µm below the diamond surface. This integration reshapes the broad dipolar emission of the targeted NV center into a tightly collimated, low‐divergence beam, achieving a 40‐fold enhancement in photon collection efficiency with an objective lens of NA = 0.07. Notably, emission is detected exclusively from metalens‐coupled NV centers, effectively filtering out background noise from neighboring defects. Second‐order correlation measurements yield g⁽^2^⁾(0) = 0.04, unambiguously confirming the generation of high‐purity single photons. This scalable, site‐specific approach addresses key limitations in low‐NA operation, opening the door to compact and fiber‐integrated solid‐state quantum photonics.

## Graphical abstract

This work addresses a fundamental bottleneck in scalable quantum photonics by enabling high‐purity single‐photon extraction from individual NV centers into ultra‐low numerical aperture (NA) optics. As low‐NA optics are essential for compact and fiber‐integrated quantum platforms, the defect‐selective metalens provides a practical and timely pathway toward real‐world quantum applications.

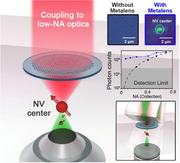

## 1 Introduction

Quantum technology is poised to revolutionize a wide range of fields, including cryptography,^[^ ^1^, ^2^, ^3^, ^4^ ^]^ communication,^[^ ^5^, ^6^, ^7^ ^]^ computation,^[^ ^8^, ^9^, ^10^ ^]^ and sensing.^[^ ^11^, ^12^, ^13^, ^14^, ^15^ ^]^ Among the diverse approaches being explored, solid‐state defect‐based quantum systems have emerged as particularly compelling candidates, offering scalability, robustness, and room‐temperature operability—key attributes for practical quantum applications. Notably, platforms such as nitrogen‐vacancy (NV) centers in diamond enable precise qubit manipulation through optical,^[^ ^16^ ^]^ magnetic,^[^ ^17^ ^]^ and mechanical^[^ ^18^ ^]^ interactions. With their exceptional spin coherence times,^[^ ^19^, ^20^, ^21^ ^]^ stable single‐photon emission,^[^ ^22^ ^]^ and strong potential for integration,^[^ ^23^ ^]^ NV centers have established themselves as a leading solid‐state platform for quantum technologies, including quantum memories,^[^ ^24^, ^25^ ^]^ quantum repeaters,^[^ ^26^, ^27^ ^]^ and magnetometry.^[^ ^11^, ^14^, ^15^ ^]^

To fully harness the potential of NV centers, it is essential to achieve both high photon collection efficiency and single‐photon purity when coupling their emission into optical systems.^[^ ^28^ ^]^ However, this is fundamentally challenged by two intrinsic properties of NV centers: a broad dipole emission pattern and a random spatial distribution within the diamond host.^[^ ^29^ ^]^ These characteristics necessitate the use of high‐numerical‐aperture (NA) optics to ensure sufficient photon collection efficiency and maintain spatial selectivity—optical systems that are typically bulky, costly, and poorly suited for integration into compact quantum devices or photonic integrated circuits. To enable widespread deployment, quantum technologies must be compatible with low‐NA optical systems, such as optical fibers and on‐chip photonic components.^[^ ^30^, ^31^ ^]^ In particular, most optical fibers employed in quantum communication and distributed quantum computing networks^[^ ^32^, ^33^, ^34^ ^]^ exhibit NA values smaller than 0.2, making conventional photon collection approaches highly inefficient. Although several strategies–such as nanodiamond‐waveguide integration^[^ ^35^, ^36^, ^37^ ^]^ and planar optics including Fresnel lenses^[^ ^38^ ^]^ and metalenses^[^ ^39^ ^]^—have been explored to mitigate this mismatch, they often suffer from severe trade‐offs, including complex alignment procedures, degraded spin coherence, and surface‐induced damage.

In this study, we present a defect‐selective metalens printing technique that enables highly targeted and substantial enhancement of photon collection from individual NV centers—even under extremely low‐NA conditions—while ensuring diamond integrity and offering excellent scalability. Using an in situ transfer‐printing process, we precisely aligned and printed high‐purity silicon dioxide metalenses directly above selected NV centers positioned 25 µm beneath the diamond surface. This approach achieves a remarkable 40‐fold enhancement in collection efficiency using an objective lens with an NA as low as 0.07. Crucially, the site‐selective approach ensures efficient collection of emission exclusively from the targeted NV center, substantially suppressing background noise from neighboring defects and yielding a high signal‐to‐noise ratio (SNR). Second‐order correlation measurements further confirm the quantum purity of the collected signal, with g^(2)^(0) = 0.04. This emitter‐specific and scalable approach seamlessly bridges high‐efficiency photon collection with low‐NA photonic integration, charting a promising path toward compact, fiber‐compatible, and scalable quantum technologies.

## 2 Device Concept

**Figure 1:**
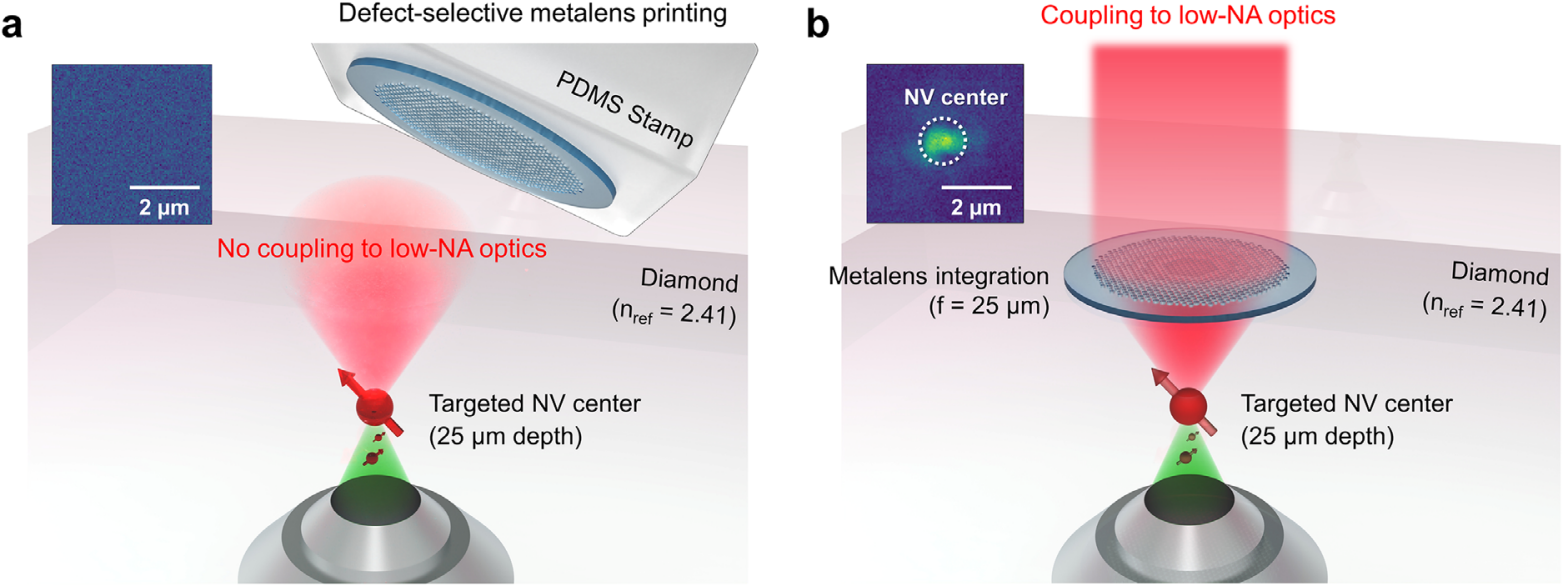
Defect‐selective metalens printing for site‐specific single‐photon collection from diamond NV centers in low‐NA optics. a) Schematic of the defect‐selective metalens printing process. A high‐purity SiO_2_ metalens with a 25 µm focal length is precisely aligned to a single NV center located 25 µm beneath the diamond surface and transferred via a PDMS stamp. b) After transfer, the metalens reshapes the NV center emission into a collimated beam compatible with low‐NA collection, forming a compact and scalable integration platform for efficient single‐photon extraction. Insets: photoluminescence (PL) maps acquired before and after metalens transfer, illustrating selective alignment and enhanced photon collection from the targeted emitter in low‐NA optics.

**Figure** **1** schematically illustrates the operating principle of our defect‐selective metalens printing strategy, which enables efficient photon extraction from NV centers into low‐NA optical systems. The process begins with the external fabrication of high‐purity dielectric metalenses, engineered with a focal length of 25 µm to match the depth of individual NV centers. In this work, we specifically target deeply embedded NVs, which, unlike shallow NVs (20–50 nm) that suffer from spectral diffusion, band bending, and surface‐trap‐induced noise,^[^ ^40^, ^41^, ^42^ ^]^ provide enhanced spectral stability and coherence. These metalenses are subsequently transferred onto the diamond surface using a soft polydimethylsiloxane (PDMS) stamp (Figure 1a), allowing precise spatial alignment between the optical axis of the metalens and the target emitter embedded within the diamond lattice. This ensures that the collected signal is predominantly derived from a single, well‐positioned NV center.

Following alignment, the printed metalens reshapes the broadband dipole emission of the NV center into a collimated output beam suitable for low‐NA collection (Figure 1b). In standard configurations, the wide angular emission of NV centers results in substantial photon loss beyond the acceptance angle of low‐NA optics. In contrast, our metalens design redirects a significant portion of this emission into the collection axis, enhancing coupling efficiency without the need for bulky external optics or precision mechanical alignment.

In addition to improving collection efficiency, this method inherently functions as a spatial filter: only NV centers that are coupled to a metalens contribute meaningfully to the detected signal. Emission from uncoupled centers remains divergent and falls outside the collection cone, effectively suppressing background fluorescence. This defect‐selective coupling significantly enhances the signal‐to‐noise ratio (SNR), enabling high‐purity single‐photon readout. The combination of precise alignment, directional emission control, and background suppression renders this device concept highly compatible with scalable quantum photonic architectures, including fiber‐based quantum networks and integrated on‐chip platforms.

## 3 Results

### 3.1 Simulation

To quantitatively assess the impact of the metalens on the emission characteristics of NV centers, we performed 3D finite‐difference time‐domain (3D FDTD) simulations. The NV center was modeled as an x‐polarized electric dipole emitter located 25 µm beneath the diamond surface, operating at the zero‐phonon line (ZPL) wavelength of 637 nm.

**Figure 2:**
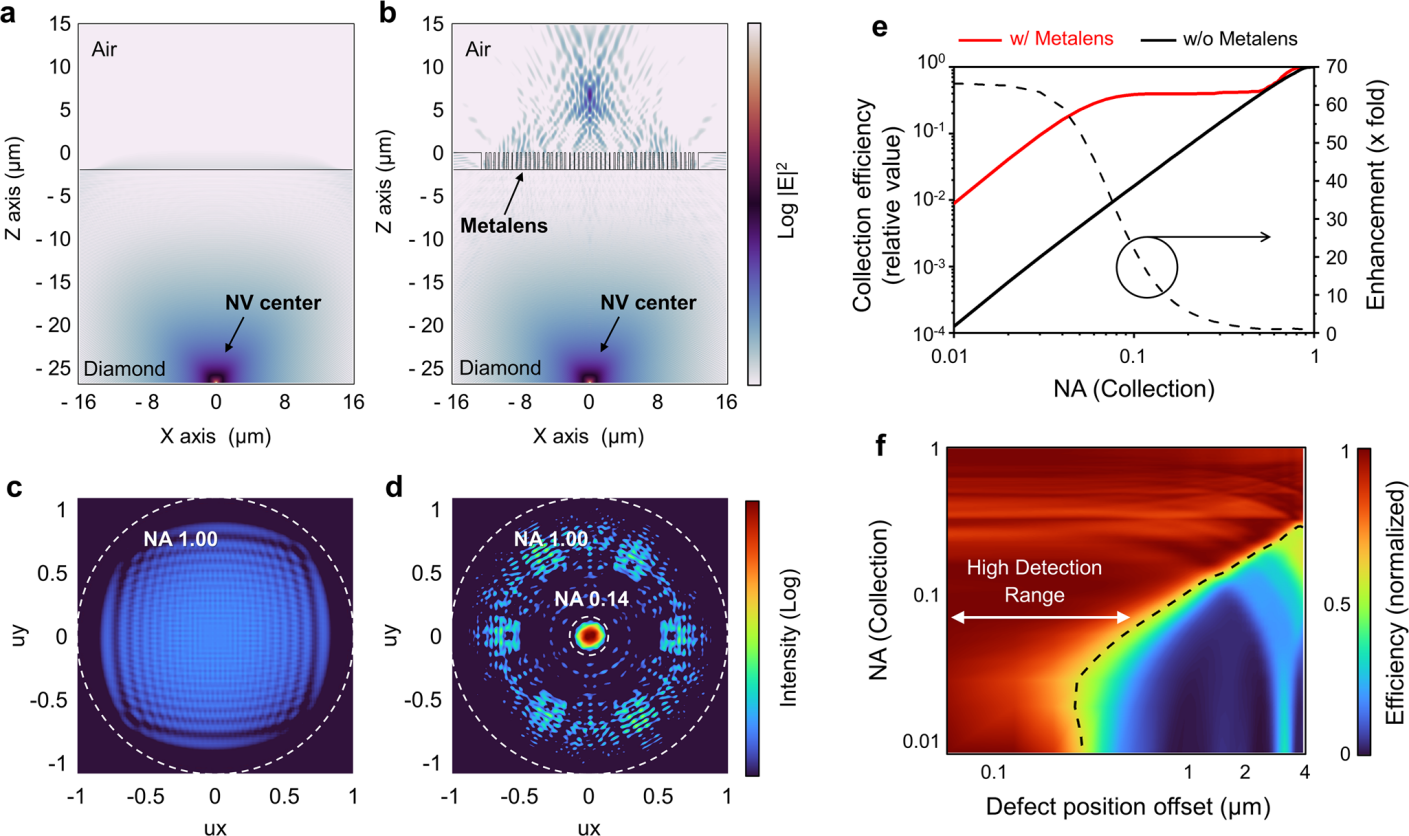
Simulations of NV center emission and collection with and without metalens integration. a, b) Simulated electric field intensity distributions (log |E|^2^) in the xz‐plane for an NV center without (a) and with (b) a metalens. The metalens collimates the emission toward the air side. c, d) Corresponding far‐field radiation patterns without (c) and with (d) the metalens, showing directional funneling of emission within NA < 0.1. e) Relative collection efficiency versus NA for structures with (red) and without (black) a metalens. The dashed curve shows the enhancement factor, with a maximum of ≈65 × at NA = 0.02. f) Simulated spatial tolerance of photon collection as a function of lateral dipole displacement and NA. The high‐efficiency detection zone (left of dashed line) narrows with decreasing NA, enabling spatial filtering of non‐targeted defects.

**Figure** **2**a,b presents simulated spatial emission profiles (log |E|^2^) in the xz‐plane, corresponding to configurations without and with the integrated metalens, respectively. The metalens comprises a 2‐µm‐thick silicon dioxide (SiO_2_) layer (n = 1.444), patterned with periodic air holes of varying diameters (170–480 nm) arranged on a square lattice with a 520 nm period. This geometry enables full 2π phase modulation at 637 nm, allowing the structure to function as a high‐NA metalens with a NA of 1.12, a physical diameter of 25 µm, and a focal length matched to the emitter depth (25 µm). The average transmission efficiency of the metalens is estimated to be 85% [see Supporting Information & Experimental Section].

As shown in Figure 2a, in the absence of the metalens, the NV center emits photons over a wide angular range due to its intrinsic dipole radiation pattern. Upon reaching the diamond–air interface, these photons undergo refraction, further increasing the angular spread and diminishing collection efficiency in low‐NA systems. The corresponding far‐field radiation pattern (Figure 2c) reveals that the emitted energy is broadly distributed across a wide range of angles, with no clear directional preference.

By contrast, when the metalens is integrated onto the diamond surface (Figure 2b), the emitted field undergoes a dramatic spatial transformation. The metalens collimates the initially divergent emission, resulting in a beam that propagates nearly vertically. This effect is clearly reflected in the far‐field intensity distribution (Figure 2d), which exhibits strong directional confinement of the emission within a narrow angular cone corresponding to NA < 0.1 (emission angle < 5.7°). These results demonstrate the ability of the metalens to efficiently funnel NV center emission into low‐NA optical systems, substantially enhancing photon collection efficiency while maintaining a compact, integrable form factor.

To quantitatively evaluate the enhancement in collection efficiency enabled by the metalens, Figure 2e presents a comparative analysis of photon collection efficiency as a function of the NA of the collection system. This analysis is derived from the far‐field intensity profiles shown in Figure 2c,d, with the collected photon flux integrated over increasing NA values. For normalization, the collection efficiency of the bare diamond structure at NA = 1.0 is set to unity, corresponding to an absolute efficiency of 6.5%, thereby enabling direct relative comparison between the two configurations. In the absence of the metalens, the collection efficiency decreases steeply with reducing NA. As NA drops from 1.0 to 0.1, the efficiency declines to ≈1% and continues to decrease for smaller NA values—reflecting the intrinsic limitations of low‐NA optics in capturing dipole emission from NV centers. By contrast, the metalens‐integrated system exhibits markedly different behavior. While its collection efficiency initially follows a similar trend down to NA ≈ 0.6, it rapidly stabilizes thereafter, maintaining a plateau ≈40% efficiency down to NA ≈ 0.08. Although a gradual decline is observed below this threshold, the efficiency remains substantially higher than that of the bare diamond across the entire low‐NA range. This distinct performance contrast underscores the effectiveness of the metalens in redirecting emission into low‐NA collection angles.

The enhancement factor, plotted as the dashed curve in Figure 2e, quantifies the relative improvement provided by the metalens. At high NA (above 0.6), the enhancement remains near unity, indicating minimal benefit where collection is already efficient. However, as NA decreases, the enhancement increases dramatically, reaching a maximum of 65 × at NA = 0.02. Notably, at NA = 0.07—the lowest value employed in our experiments—the metalens‐enabled configuration achieves a 40‐fold improvement in collection efficiency over the bare structure.

In addition to improving collection efficiency under low‐NA conditions, the defect‐selective metalens integration offers a distinct advantage in spatial selectivity, thereby enabling high SNR detection—particularly critical when multiple emitters are situated in close proximity. Figure 2f illustrates this feature by quantifying the variation in collection efficiency as a function of lateral displacement of the dipole source from the center of the metalens across different collection NA values. For direct comparison, the efficiency at each NA is normalized to unity at the optimal alignment position. A dashed curve in the plot indicates the displacement at which the collection efficiency drops to 60% of its maximum, effectively defining the spatial region over which an emitter can be efficiently detected. The area to the left of this threshold corresponds to the high‐efficiency detection zone for each NA condition.

At higher NA values (NA ≥ 0.6), the system exhibits tolerance to lateral misalignment, maintaining high collection efficiency even when the dipole is offset by more than 4 µm from the metalens center. While this suggests robust coupling over a broader spatial region, it also implies a higher likelihood of collecting unwanted emission from nearby, non‐targeted defects—ultimately degrading SNR. In contrast, as the NA decreases, the spatial window for efficient photon collection narrows significantly. Under low‐NA operation, the collection efficiency declines steeply with lateral displacement, effectively confining the detection to the targeted NV center alone. For example, at NA = 0.03, the high‐efficiency detection range contracts to ≈200 nm, whereas at NA = 0.1, it extends to ≈1 µm. This spatial filtering effect, intrinsic to the combined action of the metalens and low‐NA optics, inherently suppresses background fluorescence and enhances detection fidelity. Together, these results demonstrate that the proposed metalens‐enabled approach not only boosts photon collection efficiency but also enforces precise spatial selectivity—critically enabling high‐purity single‐photon detection in densely populated quantum emitter environments.

### 3.2 Fabrication

**Figure 3:**
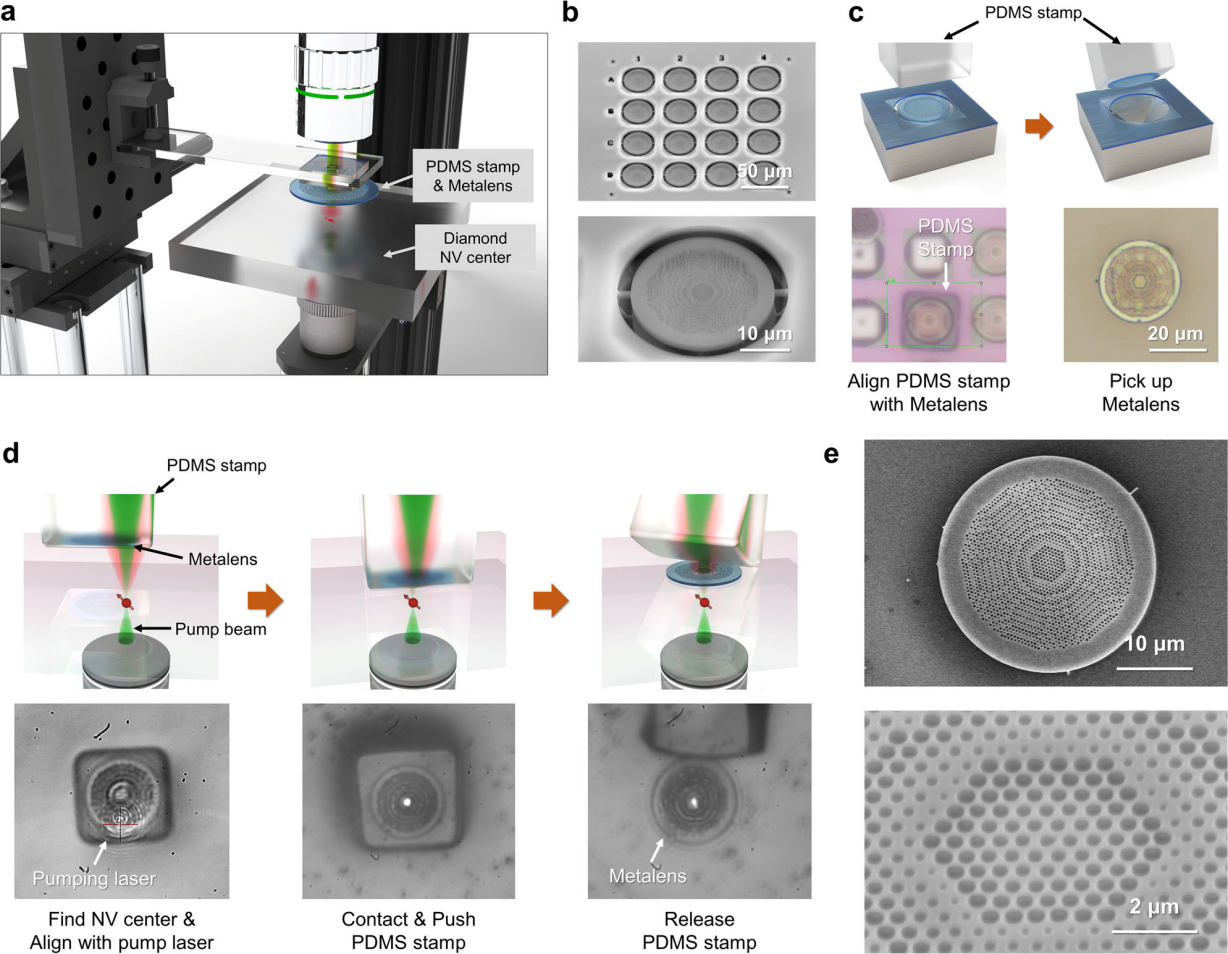
Defect‐selective metalens integration process for selective NV center coupling. a) Schematic of the in situ transfer‐printing setup with a transmission‐mode confocal microscope. A PDMS stamp carrying a metalens is aligned to the NV center optically excited from below. b) SEM images of SiO_2_ metalens arrays fabricated on a thermally oxidized silicon wafer, incorporating mechanical support frames for free‐standing transfer. c) Illustration and optical microscope images of the pickup process using a 20 µm × 20 µm × 20 µm PDMS stamp. d) Sequential steps of the in situ transfer‐printing process, showing NV center identification, alignment, and site‐selective release of the metalens. Bottom panels: Real‐time CCD images captured during each step. e) SEM images of a 2‐µm‐thick SiO_2_ metalens after transfer, confirming intact subwavelength structures and high‐fidelity transfer.

To implement the defect‐selective metalens integration strategy, we developed an in situ transfer printing technique that enables precise alignment and placement of a printable metalens directly above a target NV center in diamond. In this process, the NV center is optically excited from the bottom side, while the photoluminescence (PL) signal is collected from the top. Real‐time monitoring of the PL image during alignment allows for accurate positioning and deterministic transfer of the metalens onto the selected emitter. As shown in **Figure** **3**a, the procedure was carried out using a custom‐built, transmission‐mode confocal microscope configured to provide optical access during the transfer process. To accommodate the PDMS stamp holding the metalens, a mechanical gap was introduced between the top‐side imaging optics and the diamond substrate. A high‐NA (NA = 0.95) objective lens beneath the sample was used to tightly focus a 532 nm pump laser onto the NV center, while a 50 × long‐working‐distance objective positioned above the sample simultaneously imaged the excitation beam and the emitted PL. These optical signals served as spatial references to guide submicron alignment of the metalens, enabling selective and reliable transfer to the desired site.

To fabricate metalenses in a transferable format, a 2‐µm‐thick SiO_2_ layer was first thermally grown on a silicon wafer via furnace oxidation. Silicon dioxide was selected for its high optical quality and negligible photoluminescence background, making it ideal for detecting NV emission with minimal interference [see Supporting Information]. As shown in Figure 3b, metalens structures were patterned on the oxide surface using electron‐beam lithography, followed by anisotropic dry etching and isotropic wet etching to form a periodic subwavelength air‐hole array [see Experimental Section]. To ensure mechanical stability during transfer, each metalens was fabricated with a thin support frame at its edges, allowing it to remain suspended yet detachable using a PDMS stamp. A single 1 cm × 1 cm wafer contained ≈1000 metalens templates, with focal lengths designed to match NV centers located at different depths. For transfer, a 20 µm × 20 µm × 20 µm PDMS stamp was aligned under a microscope and gently brought into contact with the selected metalens (Figure 3c). Through van der Waals adhesion, the metalens was lifted cleanly from the substrate and subsequently transferred to the diamond surface.

Figure 3d outlines the detailed steps of the in situ transfer process. The sequence begins with optical excitation of the NV center via a 532 nm pump laser focused through the diamond from below. The resulting PL emission is continuously monitored to align the laser beam with the NV center, establishing a reliable spatial reference. The PDMS‐mounted metalens is then slowly lowered toward the diamond surface while observing the distortion of the transmitted pump beam as it passes through the lens. To enhance beam visibility, a neutral density filter attenuates the laser intensity, allowing the beam's central axis to serve as a precise optical alignment guide. Fine lateral adjustments of the PDMS stamp are made until the metalens center coincides with the beam axis. Once aligned, the stamp is gently brought into contact with the substrate. Transfer is achieved when the adhesion between the metalens and the diamond surface exceeds that between the metalens and the PDMS, allowing the lens to be cleanly released at the desired location.

Figure 3e presents scanning electron microscope (SEM) images of a 2‐µm‐thick SiO_2_ metalens following successful transfer. The high fidelity of the process is confirmed by the intact subwavelength air‐hole structures, which exhibit no visible deformation or structural damage. This pick‐and‐place procedure enables deterministic, defect‐selective integration of metalenses tailored to individual NV centers, with an average lateral alignment accuracy of ∼930 nm [see Supporting Information].

### 3.3 Measurement

Before evaluating the photon collection efficiency of NV centers integrated with the printed metalenses, we first verified the optical performance of the transferred structures. To characterize the lensing behavior, a 650 nm laser beam was normally incident from the air side onto the metalens, and the resulting light propagation within the diamond substrate was imaged using a confocal microscope. The focal properties were extracted from the 3D intensity distribution. The measured focal length was 25.3 µm—closely matching the design specification—confirming that the metalens geometry was well preserved during the transfer process. The focal depth, defined as the axial range over which the intensity exceeds half its maximum, was ≈2.39 µm. The full width at half maximum (FWHM) of the focal spot was measured to be ≈660 nm, slightly broader than the simulated value of 448 nm, likely due to fabrication imperfections or optical aberrations [see Supporting Information].

**Figure 4:**
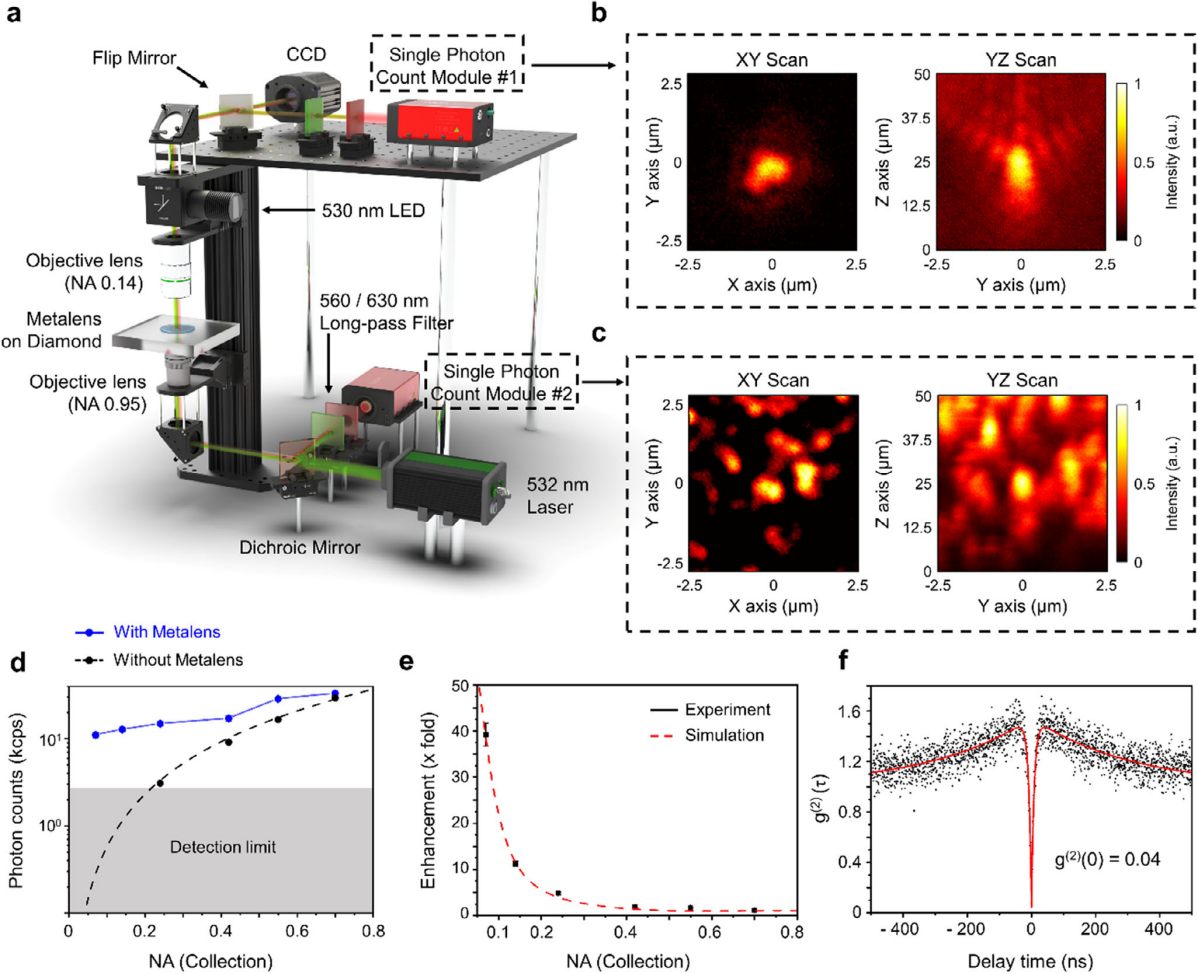
Experimental verification of site‐selective photon collection enabled by metalens integration. a) Schematic of the transmission‐mode confocal setup used for photon collection. NV centers are optically excited from below using a tightly focused 532 nm pump laser, while emitted photons are collected from above through a low‐NA objective. b) 3D PL scan (XY and YZ views) of the NV emission collected through the top low‐NA objective (NA = 0.14) after passing through the printed metalens, showing strong spatial confinement. c) PL scan collected through the bottom high‐NA objective (NA = 0.95), revealing multiple randomly distributed NV centers. d) Photon count rate versus collection NA for structures with (blue) and without (black dashed) metalenses. Without a metalens, signals fall below detection at NA ≤ 0.24; metalens integration significantly enhances collection across all NA values. e) Experimentally measured enhancement factors (black) and corresponding simulation results (red dashed) as a function of NA. A maximum enhancement of 40 × is observed at NA = 0.07. (f) Second‐order correlation measurement g⁽^2^⁾(τ) collected through the low‐NA path (NA = 0.14). The extracted g⁽^2^⁾(0) = 0.04 confirms high‐purity single‐photon emission with minimal multi‐photon contributions.

Having confirmed the focusing performance, we next assessed the photon collection efficiency of NV centers through the printed metalens and into a low‐NA optical system. These measurements were conducted using the same transmission‐mode confocal microscope employed for metalens alignment (**Figure** **4**a). On the collection side (top), objective lenses with varying NAs (0.07–0.7) were sequentially used to probe collection efficiency under different angular constraints. Excitation and back‐side collection were simultaneously performed from below using a high‐NA objective (NA = 0.95, 100 ×), allowing for direct comparison between the high‐NA (bottom) and low‐NA (top) collection paths. The diamond sample was mounted on a three‐axis piezoelectric stage, enabling high‐resolution spatial scanning. Lateral (x–y) scans covered a 5 µm × 5 µm area, while axial (z) scans spanned a 50 µm range to fully resolve the NV center's emission profile. To isolate the NV fluorescence, collected signals were filtered using a dichroic mirror and a 560 nm long‐pass filter to remove residual pump light, followed by a 630 nm long‐pass filter to suppress Raman background.

Figure 4b,c shows spatially resolved PL images acquired via the top (low‐NA, NA = 0.14) and bottom (high‐NA, NA  = 0.95) objectives, respectively. The high‐NA scan (Figure 4c) reveals a dense distribution of defects across lateral and axial directions, with average lateral spacing of ∼1 µm and axial separation up to ∼10 µm—highlighting the importance of spatially selective detection. In contrast, the low‐NA collection through the metalens (Figure 4b) yields a tightly localized PL signal centered at (x = 0, y = 0, z = 25 µm), consistent with the design parameters and simulation results (Figure 2f). The lateral detection range is ≈800 nm, and the axial resolution is governed by the ∼660 nm FWHM focal depth. Under these conditions, only the emission from the precisely aligned NV center contributes to the collected signal, while nearby emitters are strongly suppressed. Notably, even under high excitation power, no detectable signal from neighboring defects appears in the low‐NA path. Moreover, in regions without metalens coverage, no signal is detected through the top objective, further confirming that only metalens‐coupled NV centers yield measurable output. As the NA of the top objective increases, the collected signal gradually approaches that observed via the high‐NA collection path (Figure 4c) [see Supporting Information].

To quantify the enhancement in photon collection, we measured NV photon counts as a function of collection NA, both with and without the printed metalens (Figure 4d). In the absence of a metalens, photon counts dropped rapidly with decreasing NA, falling below the single‐photon counting module (SPCM) detection threshold for NA ≤ 0.24. In contrast, when a metalens was present, photon signals remained detectable across the full NA range, demonstrating its ability to redirect NV emission into narrower angular distributions. Across all conditions, the metalens consistently improved photon counts. For NA ≤ 0.42, the signal exhibited a plateau‐like behavior, consistent with the simulation results shown in Figure 2e. The dashed curve in Figure 4d represents an NA^2^ fit to the metalens‐free data, modeling the expected collection efficiency in the absence of beam shaping. This fit enabled extrapolation of reference photon counts at NA = 0.14 and 0.07, allowing for calculation of enhancement factors.

Figure 4e presents these enhancement factors, defined as the ratio of photon counts with versus without the metalens, across various NA values. The enhancement grows significantly with decreasing NA, closely following the simulated trend (red dashed line in Figure 4e). Specifically, an 11‐fold enhancement was observed at NA = 0.14, and a maximum enhancement of 40 × was achieved at NA = 0.07. Notably, the enhancement extends broadly across the 600–700 nm spectral window of NV emission [see Supporting Information]. These results clearly demonstrate the effectiveness of the defect‐selective metalens in boosting photon collection from individual NV centers under low‐NA conditions.

Finally, to verify the quantum nature of the emission collected through the low‐NA objective lens (NA = 0.14)—corresponding to the signal shown in Figure 4b—we performed a second‐order correlation measurement, g^(2)^(τ), using a Hanbury Brown and Twiss (HBT) interferometer.^[^ ^43^ ^]^ The measured correlation trace is shown in Figure 4f. A pronounced antibunching dip at zero time delay is clearly observed, characteristic of single‐photon emission from a quantum emitter. After background correction, the extracted g^(2)^(0) value of 0.04 lies well below the classical threshold of 0.5, unambiguously confirming that the detected photons originate from a true single‐photon source.^[^ ^44^ ^]^ This demonstrates that the emission collected via the metalens‐integrated, low‐NA optical system preserves its quantum characteristics. The sharpness of the antibunching dip indicates a short excited‐state lifetime, consistent with the photophysical properties of NV centers.^[^ ^45^ ^]^ Moreover, the symmetry and stability of the side lobes suggest minimal contributions from blinking or multi‐photon events, reinforcing the emitter's purity and stability.

## 4 Discussion and Conclusion

We have demonstrated a defect‐selective metalens printing technique that enables highly efficient and spatially selective single‐photon collection from diamond NV centers within low‐NA optical systems. By precisely aligning and transferring silicon dioxide metalenses onto individual emitters, we achieved a 40‐fold enhancement in photon collection efficiency at NA = 0.07, while simultaneously suppressing background signals from nearby defects. This approach not only reshapes the emission profile to match the acceptance cone of low‐NA optics, but also inherently filters the spatial origin of collected photons—ensuring high signal‐to‐noise ratios (SNR) and preserving quantum purity, as evidenced by a measured g^(2)^(0) = 0.04.

Importantly, the method offers a scalable and fiber‐compatible solution that obviates the need for bulky high‐NA optics, paving the way for seamless integration into compact, chip‐scale quantum platforms. The non‐invasive and flexible nature of the printing‐based fabrication and alignment process further broadens its applicability to a variety of defect‐based quantum emitters beyond diamond NV centers. A key advantage is efficient single‐photon collection with low‐NA optics, opening opportunities that are otherwise impractical with conventional high‐NA objectives. This capability expands the practicality of defect‐based solid‐state quantum systems in several areas: (i) quantum communication and networking, through direct fiber integration for plug‐and‐play single‐photon sources in quantum key distribution (QKD) and entanglement distribution; (ii) cryogenic quantum technologies, by avoiding the complexity of high‐NA objectives in cryostats and enabling simplified, stable operation; (iii) quantum sensing in constrained environments, such as semiconductor inspection and bio‐sensing, where compact fiber‐integrated probes can be deployed in situ; and (iv) scalable integration and packaging, where the compact, non‐invasive nature of the metalens bridges proof‐of‐concept demonstrations to deployable quantum modules.

Future developments could focus on automating the alignment and transfer processes to improve throughput and precision, as well as integrating printed metalenses into on‐chip photonic waveguides to enable hybrid quantum circuits. Furthermore, dynamic or tunable metalens architectures could be explored to realize active beam shaping or multiplexed control of multiple emitters.^[^ ^46^, ^47^ ^]^ Extending this approach to cryogenic environments and alternative material platforms—including SiC,^[^ ^48^ ^]^ GaN,^[^ ^49^ ^]^ or 2D systems^[^ ^50^ ^]^—could further broaden its impact across quantum communication, sensing, and information processing.

## 5 Experimental Section

### Design and Simulation of Metalens

To engineer efficient coupling of NV center emission into low‐NA optics, dielectric metalenses were designed, capable of tightly focusing emitted photons from targeted quantum defects. The phase profile of the metalens was derived based on Huygens’ principle, ensuring that all transmitted wavefronts converge coherently at a designated focal point located inside the diamond. The required phase shift ϕ(x, y) at each point (x, y) on the lens surface is given by:

(1) $$\phi \left(x,y\right)=-\frac{2n\pi }{\lambda }\left(\sqrt{x^{2}+y^{2}+f^{2}}-f\right)$$

where n was the refractive index of the substrate (diamond, n = 2.41), λ was the wavelength of incident light (637 nm, corresponding to the NV center's zero‐phonon line), and f was the target focal length. This expression defines a radially symmetric phase profile that ensures constructive interference at depth f beneath the surface. The spatially varying phase delay was implemented using subwavelength air‐hole arrays patterned into a 2‐µm‐thick SiO_2_ layer (n = 1.444), allowing full 2π phase coverage. Each air‐hole diameter was mapped to a corresponding local phase delay, as determined from full‐wave simulations of unit cell transmission responses [see Supporting Information].

To evaluate optical performance, 3D finite‐difference time‐domain (FDTD) simulations of the metalens structure were performed using the calculated phase map. The metalens was designed with a diameter of 25 µm and a focal length of 25 µm, yielding a numerical aperture (NA) of 1.12. The simulations modeled x‐polarized dipole emission from an NV center positioned 25 µm below the diamond surface and verified the metalens's ability to focus incident 637 nm light at the desired depth. The simulation results demonstrated a high focusing efficiency exceeding 80%, with an average transmission above 85%. The focused spot exhibited a full width at half maximum (FWHM) of 448 nm and an intensity enhancement of over 130 × at the focal plane—indicating strong localization of the emission beam. These findings confirm that the designed metalens can effectively reshape NV center emission for optimal coupling into low‐NA optical systems.

### Fabrication of Metalens Arrays for Transfer

To enable scalable and defect‐selective coupling of NV center emission, wafer‐scale arrays of freestanding dielectric metalenses optimized for transfer using PDMS stamps were fabricated. Each 1 cm × 1 cm wafer hosts ∼1000 metalens templates with varying focal lengths and numerical apertures (NA), allowing flexible matching to NV centers located at different depths within the diamond substrate. The metalens arrays were patterned on a 2‐µm‐thick thermally grown silicon dioxide (SiO_2_) layer on a silicon wafer. The thermal oxidation process was optimized to minimize background fluorescence and achieve high optical quality for single‐photon detection at 637 nm. A 50‐nm‐thick chromium (Cr) hard mask was deposited via electron‐beam evaporation to facilitate high‐fidelity pattern transfer during etching. After sulfuric acid–peroxide mixture (SPM, H_2_SO_4_:H_2_O_2_ = 3:1) cleaning, a 350‐nm‐thick positive‐tone electron beam resist (PMMA, MicroChem) was spin‐coated onto the wafer. The metalens patterns were exposed using a modified Hitachi HR‐SEM (S‐4700) electron beam lithography system and developed in a MIBK:IPA (3:1) solution. Subsequently, the patterns were sequentially transferred into the Cr mask and underlying SiO_2_ slab via two dry etching steps: Cl_2_/O_2_‐based inductively coupled plasma reactive ion etching (ICP‐RIE, STS Multiplex ICP) for Cr, followed by CF_4_/O_2_‐based ICP‐RIE (AMAT P5000) for SiO_2_. To enable mechanical release using a PDMS stamp, thin support structures were incorporated around each metalens. For additional undercutting of the underlying Si substrate and to facilitate freestanding lens formation, a combined dry‐wet etching strategy was used. First, an SF_6_‐based capacitively coupled plasma RIE (CCP‐RIE, JVAC JV19RIE‐8AP) was used to laterally etch beneath the SiO_2_ slab, opening side‐access pathways for isotropic etching. This step significantly reduced the wet etching time required to remove the remaining Si layer, mitigating damage risk to the SiO_2_ structures. Subsequently, anisotropic wet etching using KOH (30 wt.%, 80 °C) was performed to fully remove the residual Si substrate underneath the lenses, resulting in freestanding SiO_2_ metalens arrays supported only by the surrounding microstructures. Finally, the Cr hard mask was stripped using Cl_2_/O_2_ ICP‐RIE (Oxford PlasmaPro 100), completing the fabrication process. The resulting freestanding metalens arrays exhibited high structural fidelity, making them suitable for reliable PDMS‐assisted pick‐up and transfer.

### Characterization of Metalens Performance and Alignment Accuracy

To evaluate the performance of the metalens, the focal spot profile of the metalens transferred onto a diamond substrate was experimentally measured. A collimated beam with a wavelength of 650 nm, close to the zero‐phonon line (ZPL) wavelength, was incident on the metalens, and the light propagation inside the diamond was imaged using a confocal microscope. The full width at half maximum (FWHM) of the focal spot along the axial (Z) direction was measured and fitted with a Gaussian profile to estimate the focal depth. The experimentally obtained focal depth was 2.39 µm, closely matching the simulated value of 1.47 µm, confirming the accurate positioning of the focal plane. Subsequently, the transverse focal spot profile was analyzed, revealing a lateral FWHM of ≈660 nm, slightly broader than the simulated value of 448 nm. This discrepancy was attributed to fabrication‐induced aberrations due to the high aspect ratio of the nanostructures in the metalens, as well as uncertainties in the measurement process. Nevertheless, experimental data acquired from NV centers demonstrated excellent agreement with simulation results, indicating that the metalens performs near its design specifications.

During the imaging‐based characterization of the metalens focal spot, the alignment accuracy between the focal spot and the target NV center was also assessed. Using a piezoelectric stage with sub‐nanometer resolution, It was determined that the focal spot (x, y, z) was located at (97.4, 98.4, 52.7) µm, while the NV center was positioned at (97.3, 97.9, 52.4) µm, resulting in a lateral alignment error of ≈550 nm. This error was below the diffraction‐limited resolution of a conventional objective lens. As shown in Figure 2f, such a 550 nm misalignment introduces less than 2% signal collection loss under a numerical aperture (NA) of 0.14, ensuring minimal degradation in collection efficiency and signal‐to‐noise ratio (SNR). Furthermore, alignment statistics gathered from multiple fabricated samples, as presented in Figure S7 (Supporting Information), indicate that ≈70% of the transferred metalenses exhibit alignment errors below 1 µm, demonstrating high reproducibility. With improvements in the experimental setup and alignment algorithms, further enhancement of alignment precision was expected.

### Optical Setup

The optical microscopy system used in this study, based on a confocal microscope, was designed for both locating nitrogen‐vacancy (NV) centers and evaluating the performance of the integrated metalens system. As illustrated in Figure 4a, the setup consists of two main parts: (1) a custom‐built reflection‐mode confocal microscope for identifying the position of targeted NV centers, and (2) a custom‐built transmission‐mode confocal microscope for characterizing the performance of the metalens. To obtain confocal images and determine the positions of NV centers in the diamond sample (CVD‐grown quantum diamond, ElementSix), a 3‐axis piezoelectric stage (P‐562.3CD, PI) was used to enable precise scanning. NV centers were excited using a Laser Diode (LD)‐pumped solid‐state laser (MGL‐FN‐532, CNI Laser), which was tightly focused onto the diamond through a 100 ×, NA 0.95 objective lens (100x/0.95 BD DIC M27, ZEISS). Photons emitted from NV centers were collected using objective lenses positioned on both the reflection and transmission sides of the setup. On the transmission side, the microscope was equipped with long‐working‐distance objectives (Mitutoyo), adjustable in both magnification and NA, to facilitate accurate post‐characterization after the transfer printing process. This configuration enabled quantitative measurement of photon collection enhancement induced by the metalens under an identical optical path. Collected emission signals were spectrally filtered using a dichroic mirror and a 560 nm long‐pass filter to remove the pump laser. Background noise from first‐ and second‐order Raman scattering in the diamond lattice was further suppressed using a 630 nm long‐pass filter. The filtered photons were then coupled either into a multi‐mode optical fiber (M14L, Thorlabs; core diameter: 50 µm, NA: 0.22) or passed through a free‐space mode filter before being directed to a single‐photon counting module (SPCM‐AQRH‐W1, Excelitas).

This optical system was integrated with a transfer printing setup using a PDMS stamp to align and transfer the metalens onto the diamond surface. A transfer station was placed on the transmission side of the microscope, consisting of a two‐stage alignment system. Horizontal alignment between the diamond and PDMS stamp was achieved using a two‐adjuster kinematic mirror mount (Thorlabs), while fine alignment between the NV center and the metalens was performed using an XYZ translation stage (PT3A, Thorlabs), differential adjusters (DM12, Thorlabs), and a motorized vertical actuator (ZST225B, Thorlabs). To facilitate alignment during the transfer process, an incoherent LED light source (530 nm, M530L4, Thorlabs) was used to illuminate the metalens and PDMS from below. A CCD camera (Grasshopper3, Teledyne) mounted on the transmission side captured real‐time images of the NV center and the metalens for accurate overlay. Depending on the measurement mode, the optical path was switched between the SPCM and CCD using a motorized flip mirror (MFF101, Thorlabs).

## Conflict of Interest

The authors declare no conflicts of interest.

## Supporting information



Supporting Information

## Data Availability

All data supporting the findings of this study are included in this article and its Supporting Information. Additional data are available from the corresponding author upon reasonable request.
