# Supplementary material for: High‐Purity Single Photon Extraction from Diamond Nitrogen‐Vacancy Centers in Low‐Numerical‐Aperture Optical Systems via Defect‐Selective Metalens Printing
Source: Small. 2025 Nov 2;21(50):e10745. doi: 10.1002/smll.202510745 (PMC12710196; doi:10.1002/smll.202510745)
Supplement: Supplementary file 1 — Supporting Information [file SMLL-21-e10745-s001.pdf]

NANO · MICRO
small

Supporting Information

for *Small*, DOI 10.1002/smll.202510745

High-Purity Single Photon Extraction from Diamond Nitrogen-Vacancy Centers in Low-Numerical-Aperture Optical Systems via Defect-Selective Metalens Printing

Minseok Jeon, Moohyuk Kim, Nu-Ri Park, Yeeun Choi, Junghyun Lee, Chulki Kim, Sang-Wook Han, Dongyeon Daniel Kang*, Seung-Woo Jeon* and Myung-Ki Kim**

Supporting Information

High-purity single photon extraction from diamond nitrogen-vacancy centers in low-numerical-aperture optical systems via defect-selective metalens printing

Minseok Jeon†, Moohyuk Kim†, Nu-Ri Park, Yeeun Choi, Junghyun Lee, Chulki Kim, Sang-Wook Han, Dongyeon Daniel Kang*, Seung-Woo Jeon*, Myung-Ki Kim**

†These authors contributed equally: Minseok Jeon, Moohyuk Kim

Minseok Jeon, Moohyuk Kim, Nu-Ri Park, Yeeun Choi, Sang-Wook Han, Myung-Ki Kim
KU-KIST Graduate School of Converging Science and Technology, Korea University, Seoul 02841, Republic of Korea

Minseok Jeon, Nu-Ri Park, Yeeun Choi, Junghyun Lee, Chulki Kim, Sang-Wook Han, Dongyeon Daniel Kang, Seung-Woo Jeon, Myung-Ki Kim
Center for Quantum Technology, Korea Institute of Science and Technology, Seoul 02792, Republic of Korea

Junghyun Lee, Sang-Wook Han, Dongyeon Daniel Kang
Division of Nanoscience and Technology, KIST School, Korea University of Science and Technology (UST), Seoul 02792, Republic of Korea

Myung-Ki Kim
Department of Integrative Energy Engineering, College of Engineering, Korea University, Seoul 02841, Republic of Korea

*E-mail: swhan@kist.re.kr; dykang@kist.re.kr; sw_jeon@kist.re.kr; rokmk@korea.ac.kr

Contents

Supplementary Note 1: Optimization of Metastructure Design
Supplementary Note 2: Metalens Design and FDTD Simulations
Supplementary Note 3: Fabrication Procedure for Metalens Arrays
Supplementary Note 4: Selection of Thermally Grown SiO_2 for Metalens Fabrication
Supplementary Note 5: Configuration of Transfer Printing System
Supplementary Note 6: Performance Evaluation of Fabricated Metalenses
Supplementary Note 7: Measurement of Alignment Error between Metalens and NV Center
Supplementary Note 8: Characterization of Metalens-Coupled NV Centers
Supplementary Note 9: Broadband Collection Enhancement by the Printed Metalens

Supplementary Note 1: Optimization of Metastructure Design

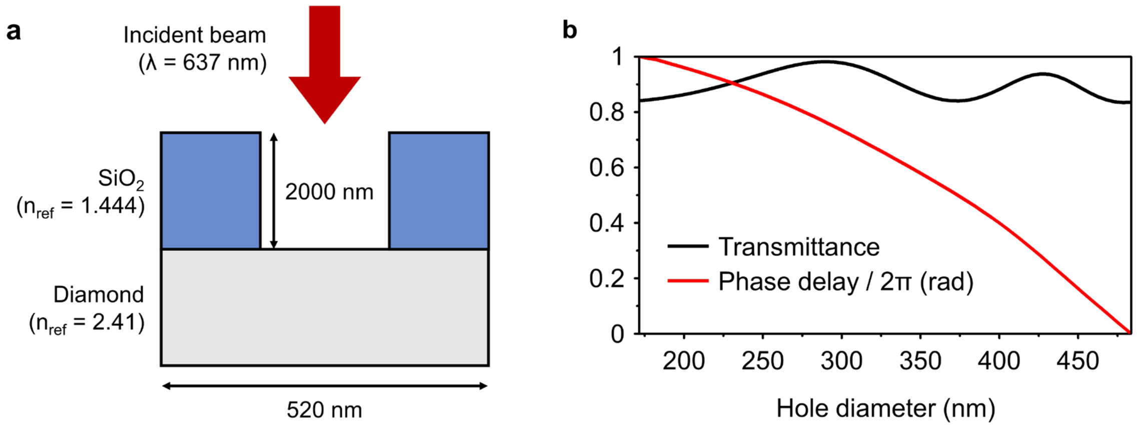

Figure S1. Optimization of metastructure design. To optimize the metastructure for operation at the zero-phonon line (ZPL) wavelength of 637 nm, we designed a hexagonally arranged SiO_2 metasurface that provides both high transmittance and full 2π phase coverage. (a) The refractive indices of diamond (n = 2.41) and SiO_2 (n = 1.444) were used as material parameters. Air holes of uniform diameter were patterned in a hexagonal lattice within the SiO_2 layer. (b) With a film thickness of 2000 nm and a lattice constant of 520 nm, simulations show an average transmittance exceeding 90% and complete 2π phase modulation as a function of air-hole diameter.

Supplementary Note 2: Metalens Design and FDTD Simulations

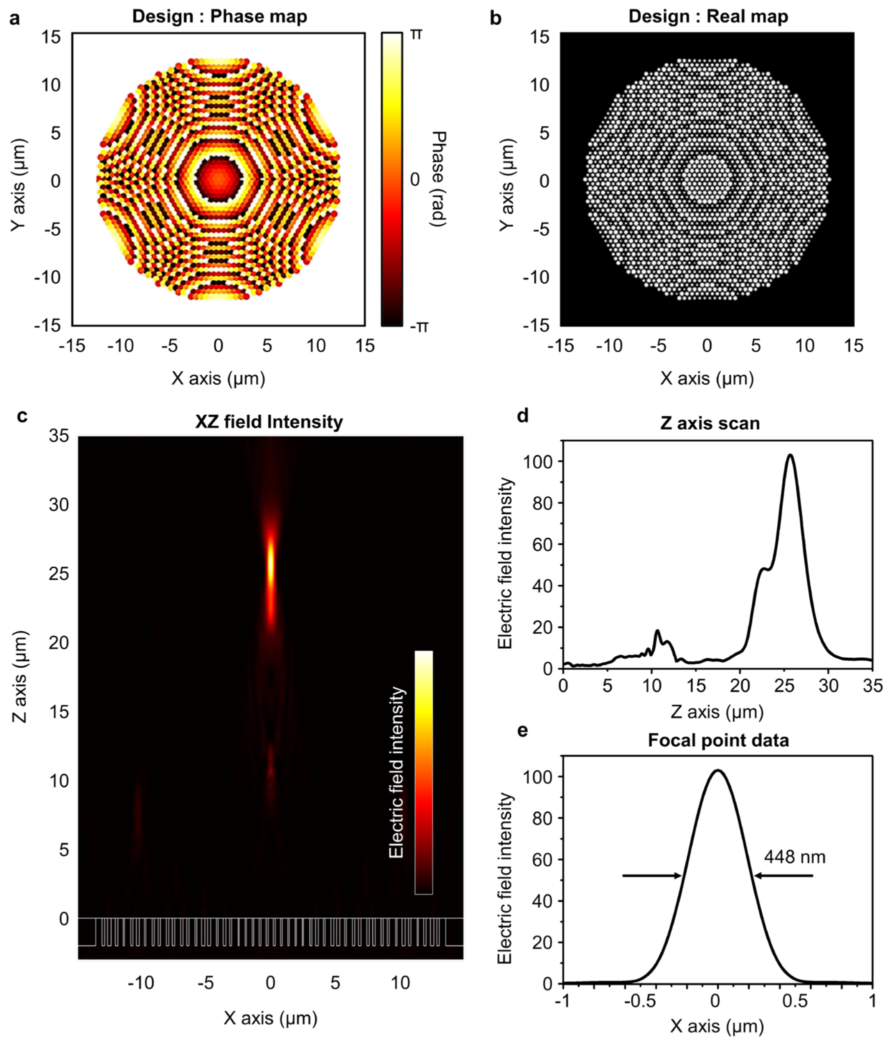

Figure S2. Metalens design and FDTD simulations. (a) Phase profile of the metalens optimized to focus emission from an NV center located 25 µm beneath the diamond surface. (b) Corresponding air-hole pattern derived from the phase modulation results in Figure S1(b). (c–e) Full-wave FDTD simulations were performed to evaluate the metalens performance by illuminating the structure with collimated light from the air side and analyzing the resulting field distribution within the diamond. (c, d) The simulations confirm that the metalens focuses light at a depth of 25 µm with a depth of focus (DOF) of ~5 µm. (e) The focal-plane intensity profile shows a full width at half maximum (FWHM) of 448 nm and an electric field enhancement factor of approximately 110 at the focal point.

Supplementary Note 3: Fabrication Procedure for Metalens Arrays

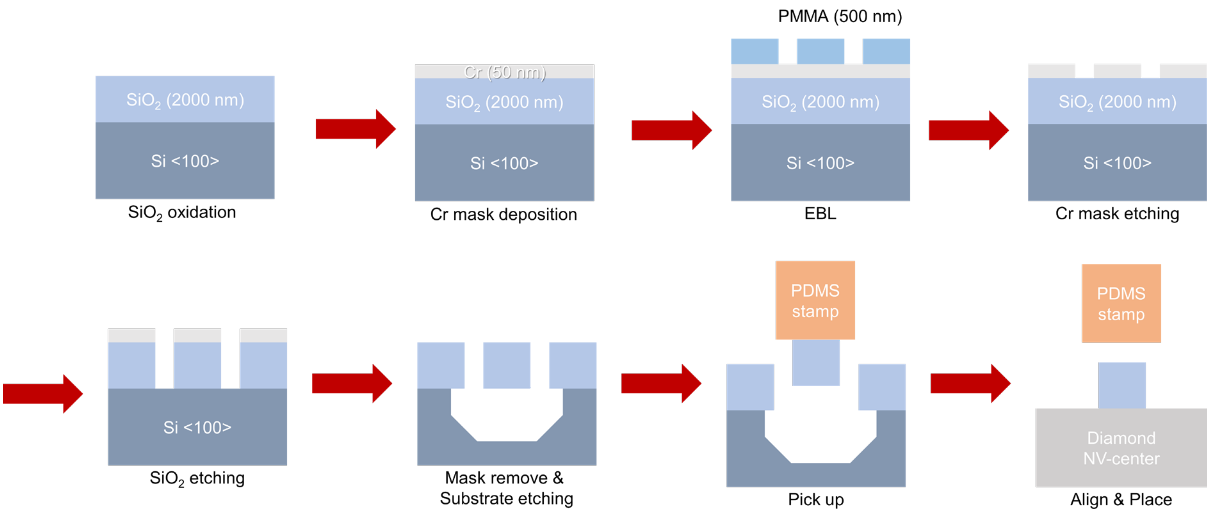

Figure S3. Fabrication procedure for metalens arrays. A thermally grown 2-µm-thick SiO_2 layer on a silicon substrate is patterned to define the metalens structures. Subsequent undercut etching of the Si substrate yields free-standing SiO_2 metalens arrays. Each array consists of a 4 × 4 arrangement of metalenses, and the full 1 cm × 1 cm substrate accommodates an 8 × 8 grid of such arrays. This modular design enables the selective retrieval of metalenses with desired focal properties for deterministic transfer onto target NV centers in diamond using a PDMS stamp.

Supplementary Note 4: Selection of Thermally Grown SiO_2 for Metalens Fabrication

In this study, we chose thermally grown SiO_2 for the transfer-printed metalenses because it offers high transmission with negligible photoluminescence (PL) background, essential for faithful detection of NV single-photon emission. By contrast, deposited oxides such as PECVD or ALD SiO_2 often incorporate hydrogen, hydroxyl groups, and structural defects that form non-bridging oxygen hole centers (NBOHCs), leading to broad PL in the visible under green excitation [S1]. Thermally grown SiO_2, formed by furnace oxidation, is stoichiometric, dense, and largely free of such luminescent defects [S2], and our control measurements confirmed that it contributes negligible background near the NV zero-phonon line (637 nm).

High-index dielectrics such as TiO_2 and Si_3N_4 are attractive for metasurface optics because they can offer low propagation loss and enable thinner metasurface geometries, but they typically exhibit defect-related PL under NV excitation—TiO_2 from oxygen vacancies [S3] and Si_3N_4 from dangling-bond or hydrogen-related states [S4]—which can significantly reduce the signal-to-background ratio and compromise single-photon purity.

We experimentally compared the PL background from metalenses fabricated with PECVD-deposited Si_3N_4 and thermally grown SiO_2 under identical conditions (Fig. S4). While the Si_3N_4 metasurface exhibited strong defect-related PL, the thermally grown SiO_2 metasurface showed negligible background emission, confirming its optical cleanliness and suitability for NV-based single-photon detection.

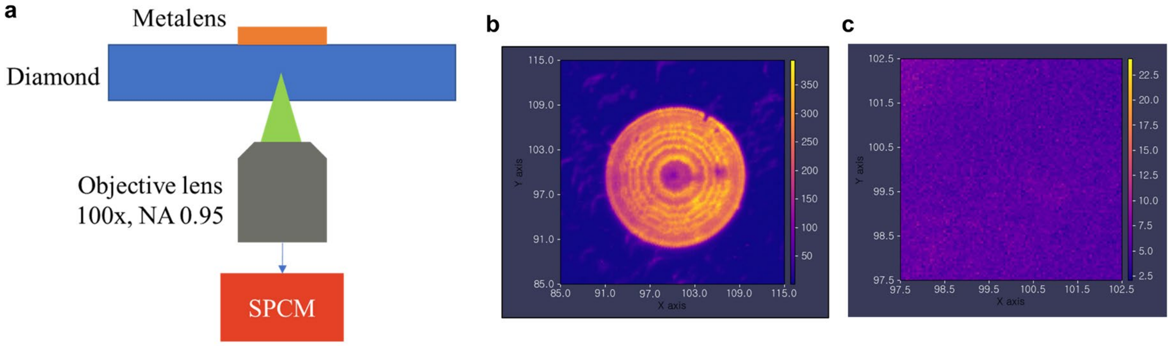

Figure S4. Comparison of PL background from different dielectric materials (a) Schematic of the PL measurement setup. PL was collected from the metasurface region using a 100×, NA 0.95 objective and detected by a single-photon counting module (SPCM). (b) PL image of a PECVD-deposited Si_3N_4 metasurface, showing strong PL emission form the metasurface. (c) PL image of a thermally grown SiO_2 metasurface, demonstrating negligible background PL.

[S1] P. F. McMillan, Amorphous Materials: Structure, Properties and Applications, Cambridge University Press, 2005.
[S2] B. E. Deal, A. S. Grove, General relationship for the thermal oxidation of silicon, *Journal of Applied Physics* 36, 3770 (1965).
[S3] J. H. Pan et al, Study of the visible-excitation luminescence of NTA-TiO_2(AB) based on the oxygen vacancies related states, *J. Lumin.* 106, 23 (2004).
[S4] J. Robertson, Defects and hydrogen in amorphous silicon nitride, *Philosophical Magazine B* 69, 275 (1994).

Supplementary Note 5: Configuration of Transfer Printing System

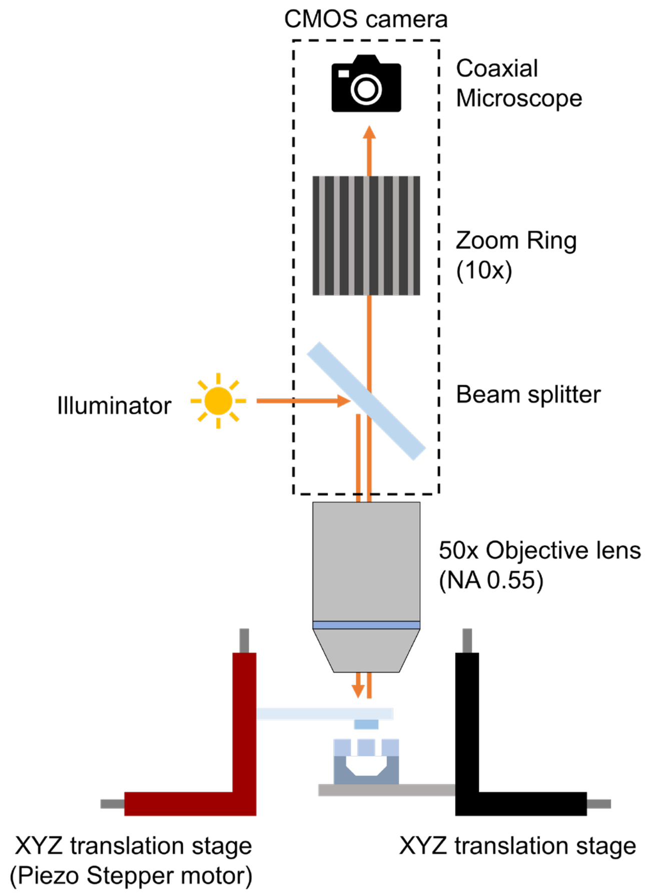

Figure S5. Configuration of transfer printing system. A 20 × 20 × 20 μm^3 PDMS stamp is used to detach individual metalenses from the prefabricated wafer and transfer them onto the diamond surface. The transfer setup comprises a coaxial microscope (CX-10C, HiRox) equipped with a 10× zoom module and a 50× long-working-distance objective lens (NA = 0.55, MY50X-805, Mitutoyo), providing up to 500× total magnification. The wafer is mounted on a manual XYZ stage (PT3A, Thorlabs) with a lockable differential adjuster (DM12, Thorlabs) for coarse positioning, while the PDMS stamp is affixed to a piezo-actuated XYZ motorized stage (9063-XYZ-PPM-M) for precise alignment and vertical motion. This configuration enables accurate pick-up and deterministic placement of the metalens onto the desired position above the NV center.

Supplementary Note 6: Performance Evaluation of Fabricated Metalenses

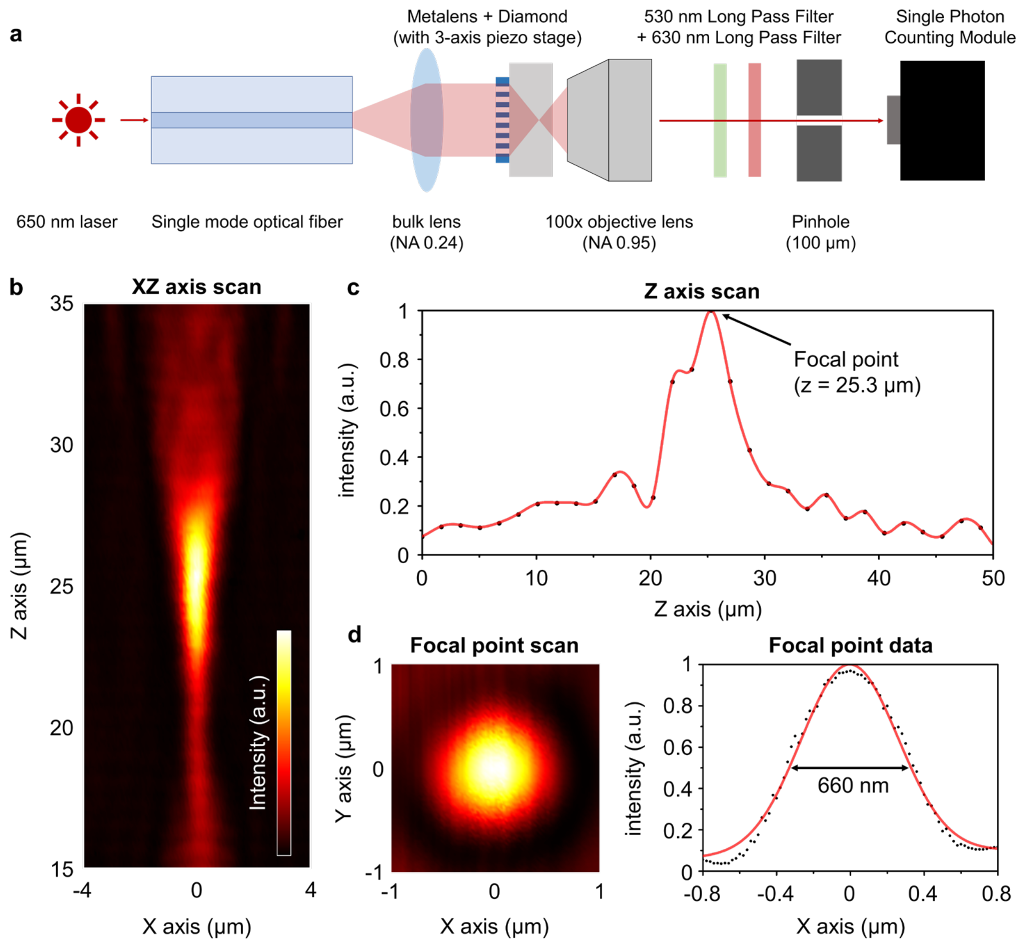

Figure S6. Performance evaluation of fabricated metalenses. (a) The focusing performance of the fabricated metalens was characterized using the confocal microscope setup shown in Figure 4(a). A 650 nm laser source was introduced at the position corresponding to the top-side SPCM of the metalens, collimated by a bulk lens (NA = 0.24), and transmitted through the metalens. The transmitted light was collected by a second SPCM beneath the metalens, while three-dimensional intensity mapping was performed by scanning the sample with an XYZ piezo stage. (b–e) Measured wavefront distributions exhibit a spatial resolution limited by the ~0.5 nm precision of the piezo stage. (b, c) An XZ-plane scan reveals a focal length of 25.3 μm and a depth of focus (DOF) of 5 μm, both in close agreement with the simulation results in Figure S2. (d, e) The focal-plane intensity profile follows a Gaussian distribution with a full width at half maximum (FWHM) of 660 nm, validating the high accuracy and focusing capability of the printed metalens.

Supplementary Note 7: Measurement of Alignment Error between Metalens and NV Center

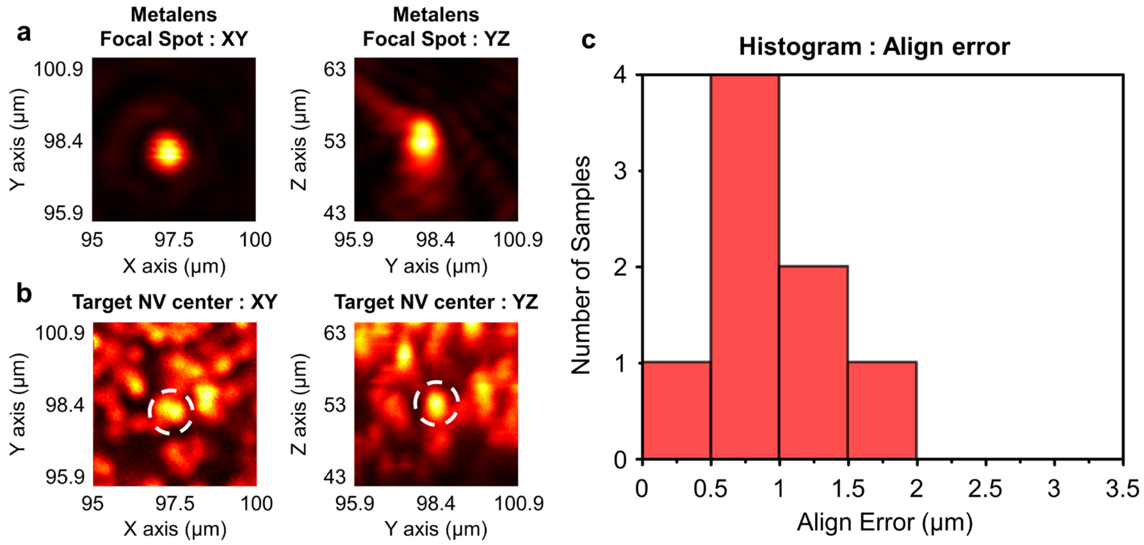

Figure S7. Measurement of alignment error between printed metalens and NV center (a, b) Alignment accuracy was evaluated by comparing the spatial coordinates of the metalens focal spot and the target NV center using a high-resolution piezo stage. Axes represent the absolute positions of the piezo scanner. (a) Confocal scans of the metalens focal region in the XY (left) and YZ (right) planes, obtained using the setup in Figure S5(a). The focal spot was located at (97.4 µm, 98.4 µm, 52.7 µm) in the X, Y, and Z directions, respectively. (b) Confocal scans of the target NV center using the setup in Figure S5(a), showing its position at (97.3 µm, 97.9 µm, 52.4 µm). The white circle indicates the target NV center. The measured displacement between the metalens focal spot and the NV center corresponds to an alignment error of approximately 550 nm. (c) Histogram of alignment errors measured across eight independent transfer attempts, showing an average lateral misalignment below 1 µm, confirming the high precision of the proposed transfer printing method.

Supplementary Note 8: Characterization of Metalens-Coupled NV Centers

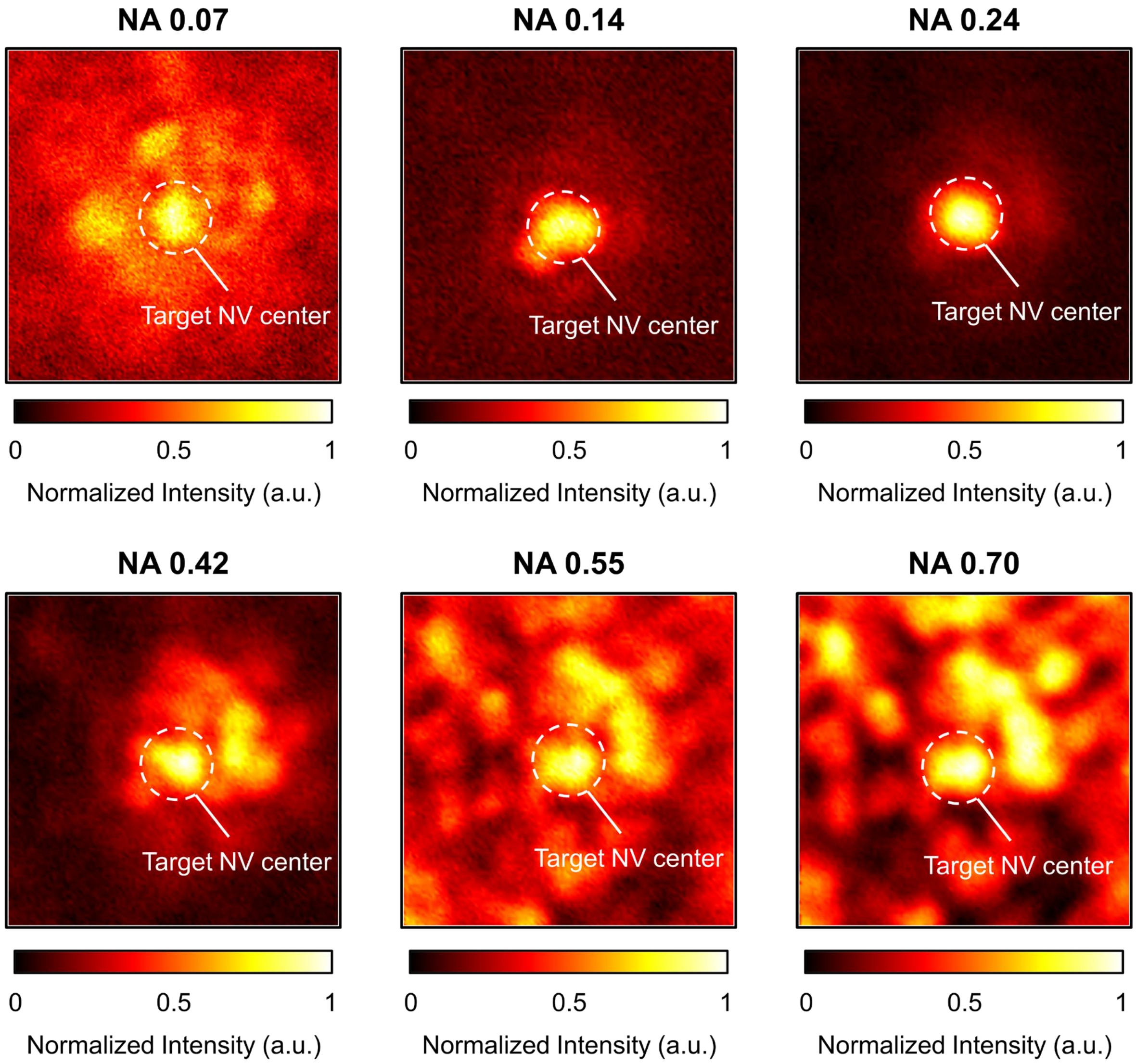

Figure S8. Characterization of metalens-coupled NV centers under varying collection NAs. Confocal PL scans of a target NV center coupled to a printed metalens, measured using objective lenses with increasing numerical apertures (left to right, top to bottom). At low NA, the metalens selectively enhances the emission from the target NV center, resulting in strong signal localization. As the NA increases, the collection cone broadens, allowing emission from adjacent non-target NV centers to enter the detection path. These results highlight the effectiveness of the metalens in enabling spatially selective photon collection in low-NA optical systems.

Supplementary Note 9: Broadband Collection Enhancement by the Printed Metalens

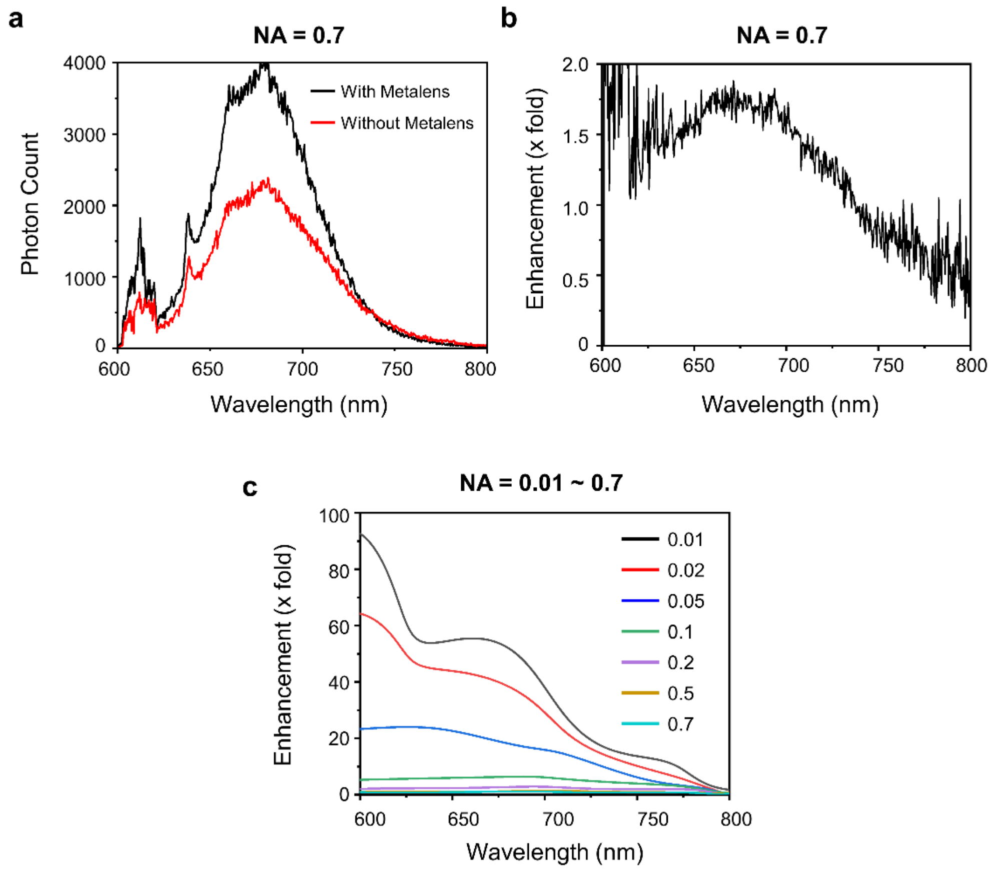

Figure S9. Broadband collection enhancement by the printed metalens (a) PL spectra of an NV center measured with (black) and without (red) the integrated metalens under a collection NA of 0.7. At lower NA values, reference spectra without the metalens could not be acquired due to insufficient photon counts, and thus spectral comparison was only feasible at NA ≥ 0.7. (b) Enhancement factor extracted from (a), showing that the metalens increases photon collection over a broad spectral range (600–700 nm). (c) Simulated enhancement spectra for different NA values (0.01–0.7), confirming that the experimentally observed broadband enhancement is consistent with theoretical predictions.
